# Major Nutritional Metabolic Alterations Influencing the Reproductive System of Postpartum Dairy Cows

**DOI:** 10.3390/metabo12010060

**Published:** 2022-01-10

**Authors:** Abdul Sammad, Muhammad Zahoor Khan, Zaheer Abbas, Lirong Hu, Qudrat Ullah, Yajing Wang, Huabin Zhu, Yachun Wang

**Affiliations:** 1 National Engineering Laboratory for Animal Breeding, Key Laboratory of Animal Genetics, Breeding and Reproduction, College of Animal Sciences and Technology, China Agricultural University, Beijing 100193, China; drabdulsammad1742@yahoo.com (A.S.); B20193040324@cau.edu.cn (L.H.); 2 State Key Laboratory of Animal Nutrition, College of Animal Sciences and Technology, China Agricultural University, Beijing 100193, China; zahoorcau@cau.edu.cn (M.Z.K.); zaheerabbas@bjtu.edu.cn (Z.A.); yajingwang@cau.edu.cn (Y.W.); 3 Faculty of Veterinary and Animal Sciences, Gomal University, Dera Ismail Khan 29220, Pakistan; qudratmahsud@gmail.com; 4 Embryo Biotechnology and Reproduction Laboratory, Institute of Animal Sciences, Chinese Academy of Agricultural Sciences, Beijing 100193, China; zhuhuabin@caas.cn

**Keywords:** dairy cow, parturition, fertility, metabolic disorders, reproductive performance, ketosis, milk fever, fatty liver

## Abstract

Early successful conception of postpartum dairy cows is crucial in determining the optimum reproductive efficiency and profitability in modern dairy farming. Due to the inherent high production potential of modern dairy cows, the extra stress burden of peri-parturient events, and associated endocrine and metabolic changes causes negative energy balance (NEBAL) in postpartum cows. The occurrence of NEBAL is associated with excessive fat mobilization in the form of non-esterified fatty acids (NEFAs). The phenomenon of NEFA mobilization furthers with occurrence of ketosis and fatty liver in postpartum dairy cows. High NEFAs and ketones are negatively associated with health and reproductive processes. An additional burden of hypocalcemia, ruminal acidosis, and high protein metabolism in postpartum cows presents further consequences for health and reproductive performance of postpartum dairy cows. This review intends to comprehend these major nutritional metabolic alterations, their mechanisms of influence on the reproduction process, and relevant mitigation strategies.

## 1. Introduction

Modern large-scale dairy farming has emerged during recent decades. There is a gross improvement in milk production, mainly attributed to intensive selection and improved nutrition. However, a constant downward trend is observed for these high-yielding cows’ reproductive performance (RP) [1,2,3]. This decline in RP may be due to increased time to the first insemination, poor exhibition of estrus behavior, increased days open, decreased success rate of artificial inseminations (AIs), and high culling rates due to poor RP [4,5]. 

There are several factors contributing to the decline in RP, including genetic factors, heat stress, and disease-related causes, to name a few [6,7,8,9]. There exists a negative correlation between milk production and reproduction, as high milk production is maintained at the expense of reproductive health [10]. Enormous nutritional consumption by the mammary system causes an alteration in the physiology of reproduction. A lactating high-yielding cow consumes a great deal of glucose and suffers from a state of negative energy balance (NEBAL) during the early postpartum period [11,12]. With the selection for high milk yield in sires used for breeding, we see a narrowing of the genetic base of major breeds throughout the world and resultant inbreeding and, together with the intensification of dairy farming, difficulties of postpartum care increased. During the post-parturient period, severe NEBAL is manifested by clinical or subclinical metabolic diseases [13]. Hence, the disease factor is the single most common cause of RP decline in dairy cows. Besides a number of infectious diseases [14], milk fever, ketosis, and other nutritional alterations contribute to RP decline [15]. Nutritional metabolic alterations during the post-parturient period affect the development and dominance of follicles on the ovaries and subsequent ovulation, while reproductive tract diseases can directly affect fertilization, embryo/fetal development, implantation, and placental development [9,16].

Thus, maintaining reproductive efficiency is an obvious challenge presented through a longer post-parturient recovery period, silent estrus, lower conception rates, and early pregnancy (within 60 d) loss [2,4,5]. These conditions and alterations mechanistically affect the body’s three major regulatory systems: the nervous system, endocrine system, and circulatory and immune system. About two thirds of reproductive disorders are encountered in the first month postpartum. Collectively, these factors directly or indirectly affect the development of follicles, the embryo/fetus, and placenta, which in turn affect cows’ RP [17,18,19]. This review aims to comprehend all these events, their interrelationships, and consequences towards fertility decline and poor reproductive performance. The main focus will be the major nutritional metabolic disorders and their complex relationship with the fertility outcomes in dairy cows. Additionally, it will elaborate on various dietary mitigation strategies helpful to improve the postpartum welfare and RP of cows.

## 2. Nutritional Characteristics, Metabolic Diseases, and Reproduction

Ruminants such as cattle ferment feed to fulfill their energy requirement and gluconeogenesis in the liver is central to lactose synthesis in the mammary gland. During the peri-parturient period, there are fluctuations in the dry matter intake (DMI). A pronounced decline in DMI is observed in the last 10 days of parturition, followed by a marked increase afterwards, but this is presumably not sufficient to fulfill the increased nutrient and energetic demand of postpartum dairy cows [20,21,22]. The post-parturient lactation peak is generally observed after 4 to 6 weeks, while the DMI peak arrives at 8 to 10 weeks. Hence, cows are in NEBAL at least 50 d postpartum. A recent study has related low prepartum DMI and energy balance with postpartum indigestion and metabolic disorders [23]. The same group of researchers associated low prepartum DMI with postpartum reproductive disorders, while postpartum reproductive problems were associated with low postpartum DMI [24]. Therefore, the phenomenon of adequate feed intake and body energy balance during the perinatal period is of immense importance for health and reproductive processes.

In addition to DMI, the body condition score (BCS) is widely used to assess the energy balance, health, and reproductive outcomes in postpartum dairy cows [25]. High prepartum BCS is associated with increased risk of postpartum metabolic problems [23], while other studies show that low prepartum BCS is responsible for prepartum metabolic and reproductive disorders [26]. The reason for this conflict in studies is essentially due to the postpartum energetic metabolism changes related to fat mobilization [27], where higher prepartum BCS (obesity) is associated with postpartum metabolic disorders and low reproductive efficiency [28,29]. Additionally, a prepartum BCS loss of 0.5 points or more could negatively affect perinatal blood Ca levels and predispose cows to the risk of ketosis and delayed conception [30]. Furthermore, high prepartum BCS was associated with lower calf body weights [31]. It is concluded that prepartum low BCS and over-conditioning (higher BCS) are both unfavorable for postpartum reproductive efficiency. The over-conditioned cows had lower mRNA levels of TNFα and higher mRNA levels of peroxisome proliferator-activated receptor gamma (PPARγ) in adipose tissues during postpartum, while the phosphorylated protein kinase B (AKT) pathway related to extensive metabolic shifts through downstream signaling of insulin in adipose tissue was also upregulated [27]. The phenomenon of high inflammatory cytokine signaling and AKT signaling pathway upregulation synergistically enhance the energetic metabolism [32,33,34]. It can be proposed that energy balance monitoring through serum metabolites can help to predict the postpartum nutritional physiology and its ultimate repercussions on reproductive performance [30,35].

Postpartum NEBAL is characterized by low blood glucose and insulin and high ketone bodies and non-esterified fatty acid (NEFA) concentrations [36,37,38,39]. However, NEBAL combined with nutritional metabolic diseases such as ketosis will delay the recovery of the uterus and normal reproductive process, leading to prolonged time to first service and increased calving intervals. In the presence of NEBAL, there is an increased incidence of nutritional metabolic disorders [40]. The literature confirms that postpartum nutritional metabolic diseases are the major causes of post-parturient reproduction disorders in dairy cows [41]. Nutritional metabolic diseases are complex “production diseases”, with a high incidence rate in modern dairy cows. In addition to the direct economic losses caused by reduced milk production, they can also have a long-term negative impact on the physiology of cows, especially their reproductive efficiency [42,43,44]. A retrospective study of 7500 perinatal Holstein cows showed that within 21 d after parturition, about 1/3 of the cows presented with at least one subclinical or clinical metabolic disease. Furthermore, the 45 d fertilization rate decreased by 7%, milk yield decreased by 14%, and the culling rate increased from 22.6% (no clinical disease) to 35.7% (one clinical incidence) and 53.8% (more than three clinical incidences) for the cows suffering with metabolic diseases [45]. Another study indicated a 26% incidence rate of ketosis during the observation period of 60 days postpartum [46]. A study covering 12 countries from four continents found that the average subclinical ketosis prevalence in the first 21 days postpartum was 24.1%, ranging from 8.3% to 40.1% [47]. Another study based on commercial dairy farm data reported a higher incidence of 56% for clinical or subclinical metabolic and reproductive diseases (ketosis, hypocalcemia, metritis, and mastitis) in the first 3 weeks postpartum [48]. 

**Figure 1:**
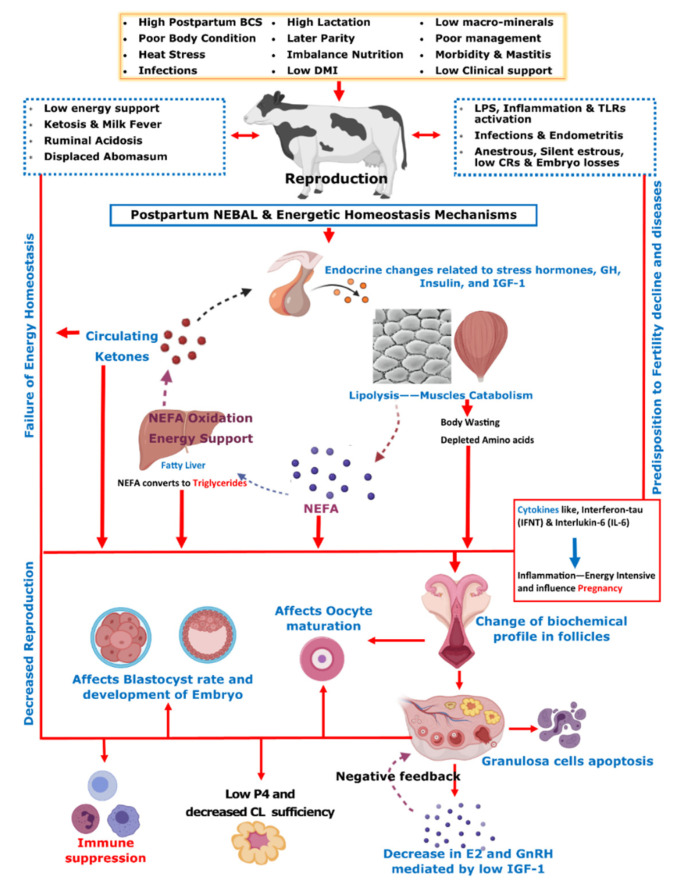
On the basis of contents discussed in the manuscript, this summary chart illustrates post-parturient metabolic alterations resulting in the decline in reproductive performance in dairy cows. The postpartum dairy cow is over-stressed due to parturition labor, lactation demands, possible exposure to heat stress, reduced dry matter intake (DMI), uterine involution, and initiation of the reproductive cycle. Due to these problems, post-parturient dairy cows usually suffer from a negative energy balance (NEBAL). NEBAL leads to endocrine and metabolic alterations initiated by low insulin, high glucose consumption, decreased insulin growth factor (IGF-1), and high growth hormone (GH) activity, leading to high non-esterified fatty acid (NEFA) response. NEFA is oxidized in the liver for energy support, leading to ketosis, and ultimately results in the development of fatty liver due to the accumulation of triglycerides (TGs). A higher prepartum body condition score (BCS) is determined as a predisposing factor for extensive mobilization of body fat reserves in the form of NEFA. Fat mobilization and protein metabolism lead to the depletion of the most required fatty acids and amino acids for reproduction and body well-being. These changes are the primary cause of secondary metabolic diseases such as hypocalcemia, ruminal acidosis, and displaced abomasum. Furthermore, they ultimately cause changes in the biochemical profile of the ovarian follicles, contained oocyte, developing embryo, corpus luteum (CL), and uterus, which ultimately result in low conception rates (CRs), whereas they also trigger endocrine changes at the pituitary–hypothalamus-ovary axis, including changes in estrogen (E2), gonadotrophins (GnRH), luteinizing hormone (LH), and progesterone (P4). Additionally, these aforementioned changes also influence the immune system of postpartum dairy cows through activation of LPS, cytokines, and Toll-like receptors (TLRs). These phenomena predispose the cows to infections and inflammatory conditions and thus contribute to the decline in CRs.

Initial post-parturient lactation is maintained at the expense of a decline in reproductive performance [10]. The mechanism involving underlying NEBAL can be explained by low insulin levels causing an abundance of growth hormones (GHs), which in turn mobilize NEFAs. However, at the same time, there is a decrease in hepatic GH receptor abundance, preventing negative feedback through IGF-1 [38,49], while the presence of NEFAs is consistently behind the low insulin via catecholamines [50]. As lipolysis helps the lactation demand, this is also the major cause of low BCS in postpartum cows. Thus, postpartum BCS is an indirect measurement of fat metabolism and correlates with the energy balance of cows [51]. These aforementioned metabolic alterations mediated by complex endocrine changes have further consequences for ovulation, oocytes, and the corpus luteum [38,49,52,53,54]. Further connections of these changes with reproductive performance include low concentrations of insulin and insulin growth factor 1 (IGF-1) causing follicular biochemical changes in ovaries and thus influencing luteinizing hormone (LH) and estradiol (E2) secretion [52,53,55]. This decrease in LH and E2 secretions in turn ultimately leads to delayed resumption of the estrus cycle [56]. On the other hand, NEBAL is also shown to be associated with low progesterone concentrations, which negatively influence the early pregnancy outcome [57,58]. Carrying the discussion further, a high concentration of blood NEFAs is shown to be negatively associated with the developmental competence of fertilized oocytes [59,60]. Generally, an increase above 0.55 mmol/L of plasma NEFA levels is regarded as a manifestation of serious postpartum NEBAL [61,62]. Postpartum NEBAL and high NEFA concentrations have shown evidence for higher levels of inflammation characterized by cytokines and Toll-like receptor (TLR) expression leading to alterations in uterine functions [63,64,65]. Furthermore, NEFA exerts cytotoxic effects at cellular levels and is attributed to immunosuppression [66,67]. As NEBAL in itself possesses implications for early reproductive recovery, it is also obvious that postpartum NEBAL can be regarded as a root cause of various postpartum production diseases. Based on the discussions in this review, Figure 1 summarizes various metabolic and endocrine mechanisms contributing to the decline in post-parturient reproductive efficiency of dairy cows. A score of mitigation strategies have been advised to minimize extreme postpartum NEBAL incidence. Dry cow management should be oriented at the feeding of energy-rich diets over the duration of 3–4 weeks prepartum, in order to support fetal growth, the decline in DMI, and peri-parturient events [68]. This phenomenon of increasing the energy content of the diet can also be supported by the facts of decreased DMI intake and rumination time during the last 3 weeks of gestation [21,24,69]. However, over-conditioning of prepartum cows is not desirable and leads to postpartum metabolic disorders [41,70]. A prepartum controlled energy diet near the calculated requirements essentially averted the risk of postpartum NEBAL [70,71]. An increased energy prepartum diet is shown to enhance fat accumulation, decrease DMI, and increase the risk of health problems in postpartum dairy cows [68,70,72]. Similarly, others have shown that an increased energy diet prepartum can lead to increased NEFA mobilization and triglyceride (TG) accumulation in the liver of postpartum cows [73], while a controlled energy diet prepartum is shown to improve the immune function of postpartum dairy cows [74]. Therefore, careful feeding management through monitoring of feed energy content and BCS assessment of prepartum cows constitute an essential key to perinatal transition success.

## 3. Fatty Liver

Fatty liver (liver lipidosis) is a secondary peri-parturient metabolic disorder with high subclinical prevalence characterized by high TG accumulation in the liver [75,76]. Behind the backdrop of a variety of peri-parturient events and NEBAL, excessive NEFA mobilization is the major cause of fatty liver, mainly due to obesity and decreased DMI of parturition dairy cows [76,77]. The liver serves as the destination for NEFAs derived from fat mobilization, where they are oxidized into acetyl-CoA, which then condenses with oxaloacetate in the TCA cycle. During states of high NEFA delivery, much acetyl-CoA diverts from the TCA cycle to ketogenesis, producing β-hydroxy-butyric acid (BHBA), acetyl-acetic acid, and acetone. Fatty liver develops when the liver uptake of NEFA exceeds the oxidation and secretion capacity of the liver, where excess NEFAs are re-esterified to form TGs in the liver and are associated with decreased metabolic functions of the liver [75,78]. High TGs are shown to alter glucose metabolism in early pregnancy and can cause pre-eclampsia [79,80]. A postpartum fatty liver incidence rate of 56% has been reported in previous studies [79,80], while the probability of pregnancy is shown to be 30% lower for cows with higher contents of liver TGs [80]. The presence of inevitable NEBAL-associated metabolic changes and inflammatory condition-associated increases in cytokine levels are the major culprits behind the development of fatty liver [27,81,82]. As a direct outcome of NEBAL, fatty liver causes inhibition of gluconeogenesis, promotes ketosis, and decreases immune functions [77,83]. As fatty liver compromises liver function, it directly influences the reproductive system through the decline in NEFA oxidation as an energetic source, and indirectly influences the RP through the disposition of postpartum cows to ketosis and allied complications.

Regarding the occurrence of NEBAL and mobilization of NEFA, a manifestation of the fatty liver leading to ketosis, it is important to discuss ameliorating approaches. A variety of fatty acid supplementations have been used to try to modulate high NEFA responses during postpartum NEBAL [84]. Dietary supplementation of 3% to 4% vegetable oil has been associated with the improvement of postpartum reproductive performance [85,86]. A study shows that feeding saturated fatty acids increased the risk of fatty liver, while feeding unsaturated fatty acids sources such as flaxseed (3.3% and 11.0% of the dry matter in prepartum and postpartum diets, respectively) during the transition period could be a useful strategy to increase liver concentrations of glycogen and decrease liver TGs after calving [87]. Prepartum feeding of canola and sunflower (8% of DMI) is shown to have positive effects on energetic metabolism in postpartum cows, however, the transition from prepartum to lactation appeared to be the main driver of changes in energetic metabolism [88]. An interesting study shows that postpartum hydrogenated TG supplementation exerted more positive effects on hepatic lipid and glycogen metabolism than palm oil supplementation [89]. Contrary to high prepartum fat supplementation, postpartum high fat feeding is shown to increase TGs and decrease cholesterol levels, while switching from high prepartum fat to low postpartum fat supplementation increased cholesterol levels, feed intake, and milk production [90]. Feeding of unsaturated fatty acids (UFAs) is shown to improve the ovarian follicle biochemical profile, improve progesterone (P4) during the luteal cycle, and modulate prostaglandins during early pregnancy [5,91,92]. Polyunsaturated fatty acids (PUFAs), such as fish oil-based rumen protected supplementation, are the best in the class of dietary fats, which are shown to improve ovarian follicle growth, increase insemination success, and are helpful in the prevention of pregnancy losses [93,94]. Peroxisome proliferator activated receptors (PPARs) have an integral role in embryonic development and early pregnancy through lipid metabolism support. Due to their reported activation through NEFA and other fatty acids of dietary origin and subsequent coordination of lipid metabolism, studies have tried to enhance their expression and found that dietary fatty acids are helpful in this regard [33,95,96]. In conclusion, several nutritional supplementation-based mitigation strategies have been devised to avert the risk of fatty liver. However, a clear direction of the type of fat supplementation, its timing, and the duration of supplementations may constitute a future avenue of studies in complex in vivo trials.

## 4. The Impact of Ketosis on Reproductive Efficiency of Dairy Cattle

Ketosis refers to a nutritional metabolic disease of the blood circulation in which ketone bodies exceed normal physiological levels, and the excessive accumulation of fatty acids in the liver is the main cause of ketosis. Ketosis is a common metabolic disease around the post-parturient period [97], and later parity cows are more susceptible to the incidence of ketosis [98]. Predisposing factors of ketosis are similar to the ones causing postpartum NEBAL, NEFA mobilization, and hepatic lipidosis, discussed in the earlier sections. The liver oxidizes this NEFA and produces ketone bodies, and circulating NEFA and ketone bodies assist in overcoming the dairy cows’ energy requirements when suffering from NEBAL [39,53,99,100]. The more severe the NEBAL in the post-perinatal period of cows, the more NEFAs are transferred to the liver, leading to rapid increases in concentrations of ketone bodies such as BHBA, and eventually leading to different degrees of ketosis in cows. Since BHBA concentrations in the serum can directly reflect the extent of ketosis, they are often used as an important indicator for the diagnosis of ketosis.

Studies have found that the incidence of ketosis in the first 2 postnatal months is 2% to 15%. However, the incidence of subclinical ketosis during the same period could be as high as 40% to 60%. In addition, studies show that high-yielding cattle may have higher incidences of ketosis, due to more severe NEBAL in the post-parturient period [13,101,102]. Ketosis causes a decrease in the quantity and quality of milk, besides a decline in fertility in dairy cows. In one study, the economic losses of each cow with subclinical ketosis were averaged to be about USD 51, and the economic losses of clinical ketosis were about USD 232 per cow, while the long-term economic losses of subclinical ketosis in later parity cows can even reach USD 213 per head [103,104]. It affects the ovarian activity, the uterus and fallopian tubes, fertilization, and early and late embryo development [105,106,107,108,109]. A study considering the prenatal NEFA concentration and its relationship with metabolic hormones showed that cows in the prenatal high NEFA group were less likely to resume the estrus cycle than cows in the low NEFA group, suggesting that high concentrations of NEFA inhibited the recovery of postpartum ovulation [15].

### 4.1. Ketosis and Ovarian Dynamics

Postpartum ovarian follicle recovery or the initiation of development is the basis of the cow’s normal reproduction resumption. With the development of follicles, the amount of E2 secretion is increased, causing signs of estrus and triggering the LH surge for ovulation. Therefore, it directly determines the first postpartum service and the fertilization rate. Studies show that cows with subclinical ketosis delayed the exhibition of estrus and the duration was relatively shorter when compared to healthy cows. This suggests that ketosis affects the development of follicles and the normal synthesis and secretion of E2 in cows after calving [110]. NEFA accumulates in the follicle fluid, changing the composition of its metabolite profile, directly affecting the energy metabolism level of granulosa cells and causing apoptosis [111]. Studies show that, when compared with healthy cows, there is a decrease in time to first ovulation and 60 d postpartum conception rates in the cows with postnatal high blood NEFA and postpartum subclinical ketosis, respectively [105,112]. The high concentration of NEFA and BHBA in the blood circulation of diseased cows decreases and degrades the insulin-like growth factor (IGF-1) [113]. IGF-1 has a direct impact on the GnRH activity and LH activity, causing reduced E2 secretion. This phenomenon leads to failure of the follicular dominance and subsequent ovulation, delaying the time to first ovulation. In contrast, studies show that if follicles ovulate, the embryo quality remains poor. A decrease in IGF-1 concentration also leads to a decrease in progesterone secretion, and in that case, if initial pregnancy is confirmed, there is a greater chance of early embryonic death. Therefore, ketosis has long-lasting effects on follicles, ova, and embryonic development [59,112]. In conclusion, ketosis has a longer cycle of effects on cow reproduction and may involve follicle development to embryonic implantation.

### 4.2. Ketosis Association with Oocyte Maturation and Implantation

Metabolic disorders are manifested through changes in metabolites in blood circulation; therefore, the composition of follicle fluid is altered. Studies have confirmed a significant increase in BHBA and NEFA concentrations in ketosis cow follicle fluid [111]. A comprehensive study found high NEFA and BHBA, but lower glutathione, in the ovarian follicle fluid of cows suffering from liver diseases. Moreover, BHBA supplementation affected the concentration-dependent decrease in oocyte maturation, while the effect on blastocyst rate was significant [114]. In the presence of high NEFA, similar results are shown in cattle and human studies, where the follicular dominance and time to ovulation and blastocyst rate were reduced, respectively [115,116]. 

Early embryo development and implantation are the most complex events in developmental biology. Fertilized oocytes journey through the development and blastocyst stage towards the preimplantation stage. The development of a preimplantation embryo is of paramount importance, as it is a prerequisite for maternal identification, uterus implantation, and gestation. Studies have shown that the lipids nourish the embryo, which is regulated by PPARγ. In this context, PPARγ activity is essential for the extension and survival of embryos, and changing the concentration or composition of fatty acids in tissue fluids can potentially alter the development of fertilized oocytes [117]. An increase in the concentration of NEFA in blood, follicles, and in the uterus during ketosis may affect the elongation and survival of preimplanted embryos by affecting the metabolism of fatty acids [44,118]. A correct balance between inflammatory and anti-inflammatory reactions in the uterus is required for early embryo attachment and implantation, and an active immune system regulates this delicate balance [119,120]. Immune suppression in cows suffering from ketosis is also an important factor affecting embryo implantation. It can increase postpartum cows’ susceptibility to bacterial pathogens due to low resistance and thus increase the risk of uterine inflammation and endometritis [121,122]. Therefore, ketosis may affect the implantation of early embryos through alteration of the endometrial fatty acid metabolism and immune suppression, resulting in decreased fertility in cows. Given the importance of ketosis, feeding high-quality concentrates accompanied with prevention of over-feeding in later gestational stages can avert the occurrence of NEBAL and associated metabolic diseases [68,123]. Feeding of a glucogenic diet comprising starch in early lactation stages and lipogenic diets in later stages of breeding can improve the reproductive process [124].

## 5. The Impact of Hypocalcemia (Milk Fever) on Reproductive Efficiency of Dairy Cattle

Postpartum cows face the challenge of increasing demand for minerals, especially calcium, to support early lactation [125,126]. Increased prolactin, parathyroid hormone (PTH), and calcitriol levels are mainly involved in calcium homeostasis during the perinatal period [127,128,129]. Studies have shown that serum calcium drops by 9 h before and returns to the normal range by about 72 h after birth [130]. Lactation is the main cause of low blood calcium in cows after parturition, as the demand increases rapidly and calcium consumption is higher than the absorption, so hypocalcemia occurs. The incidence of hypocalcemia is high in smaller breeds with high milk production such as Jerseys [127]. BCS ≥ 3.00 and summer calving have been associated with higher risk of subclinical hypocalcemia on day 1 of parturition, where day 1 incidence cows took 32 days longer to get pregnant than normo-calcemic cows [131]. Most high-producing cows develop some degree of hypocalcemia (SCH) on the first day after calving, but only if the blood calcium concentration drops to a certain level, which can disrupt neuromuscular function and is detrimental. Hypocalcemia is divided into subclinical (total calcium concentration of 1.4 to 2.0 mmol/L) or clinical (total calcium concentration < 1.4 mmol/L), accompanied by mental restlessness, anorexia, mild paralysis, and even death. Compared to primiparous cows, later parity cows are relatively more susceptible to developing clinical symptoms of milk fever [132,133]. Hypocalcemia is a risk factor for causing ketosis, displaced abomasum, mastitis, retained placenta, and uterine prolapse [134] and thereby presents a greater chance of culling. The variable average incidence of milk fever is reported by different studies, ranging from 7.2% [135] to 21% [136]. 

Studies show that hypocalcemia negatively affected the recovery of ovarian function during the voluntary waiting period, reduced the rate of pregnancy after the voluntary waiting period, and reduced the pregnancy rate after first service [137]. Cows with chronic subclinical hypocalcemia are shown to have even more pronounced impaired reproductive function [137]. Subclinical hypocalcemia diagnosed on postpartum day 1 is shown to be responsible for low fertility rates, while diagnosis at both day 1 and day 7 was related to health issues in dairy cows [131]. Retained fetal membranes and uterine inflammation are typically associated with subclinical hypocalcemia [138,139]. Similarly, another study found that hypocalcemia had a negative effect on reproductive performance through a significant increase in the time to the first conception after birth and a higher risk of culling. However, they found that hypocalcemia had no effect on first postpartum service; this could be attributed to the husbandry practices [135]. Contrary to this, higher postpartum serum calcium concentrations are associated with higher serum total cholesterol, albumin, and glucose concentrations, a lower rate of placental retention, and clinical endometritis [140]. Abnormality in calcium homeostasis is also determined as a contributing factor towards arrested follicular development and acyclic ovaries [141]. About 50% of freshly calved multiparous cows are believed to suffer from hypocalcemia [142,143]. Together with blood fatty acid profile, serum calcium levels are useful to predict the incidence of displaced abomasum [144]. Given its association with the incidence of other reproductive diseases, the post-parturient blood calcium level is of paramount importance. Several studies show that blood calcium levels after 24 h of calving are positively associated with metritis [145]. 

Clinical milk fever cases should be treated with an intravenous infusion of calcium gluconate (23%, 500 mL = 10.8 g of calcium) [146]. Prepartum feeding of a diet with a negative dietary cation–anion difference (DCAD) produces a mild metabolic acidosis in prepartum cows which is demonstrated to be helpful to avert the risk of developing milk fever [134]. From −21 d prepartum, reducing DCAD (starting from −7.4 mmol/100 g to −16 mmol/100 g of dry matter) is shown to avert the risk of hypocalcemia and associated reproductive problems [147,148,149,150]. An alternative to negative DCAD is the feeding of low-calcium diets (<20 g per day) prepartum, which can improve calcium homeostasis [127,151]. Low circulating magnesium concentrations are associated with low blood calcium levels in dairy cows [152,153]. Phosphorous status also influence a cow’s ability to regulate calcium concentrations in the blood around parturition, where reduced prepartum dietary phosphorus intake can increase perinatal circulating calcium concentrations [154]. High-calcium forage such as alfalfa should be exchanged for low-calcium forages such as corn silage to improve calcium levels postpartum [155]. Dietary zeolite (sodium aluminum silicate) during the last 2 weeks prepartum improved the circulatory calcium levels [134,156]. Serotonin (5-hydroxytryptamine, 5-HT) has been shown to improve calcium homeostasis in postpartum dairy cows [157]. The combined use of negative DCAD (−55 v. +14 mmol/kg DM) with the supplementation of 1 mg/kg body weight of 5-hydroxy-l-tryptophan (5-HTP) prior to parturition resulted in additional increases in calcium concentrations compared to negative DCAD or 5-HTP alone [158]. These major mitigation approaches discussed here can be effectively employed to decrease the risk of hypocalcemia in dairy cows.

## 6. Ruminal Acidosis and Reproductive Efficiency of Dairy Cattle

The rumen contains a dense and diverse microbiota involved in digestion, and dietary organic compounds are hydrolyzed and fermented into volatile fatty acids (VFAs) and gases. These VFAs are absorbed into circulation and support around 70% of the energy supply. The concentrate ratio of the feed is increased to overcome postpartum lactation demands and NEBAL; the irrational increase in this concentrated feed can easily lead to rumen acidosis in postpartum cows. Ruminal acidosis is a nutritional metabolic disease in the high-yielding dairy cow that affects normal fermentation due to the decline in rumen pH, caused by feeding energy-rich diets. The post-calving period is high metabolic activity time, and cows’ ability to adapt is over-stressed [39]. In general, ruminal acidosis can be divided into subacute acidosis (pH is 5.2 to 5.6) and acute acidosis (pH below 5). Subacute acidosis is characterized by repeated bouts of pH decline, while acute ruminal acidosis is mainly characterized by lactic acid accumulation with a persistent pH drop and clinical manifestations [159,160]. The incidence rate of subacute acidosis ranges from 11% to 26% [161], and it can severely impact feeding behavior and DMI, milk quality and yield and cause indigestion and reproductive incapacity due to nutritional deficiency [162,163,164]. Ruminal acidosis affects the ruminal microbiome and causes endotoxemia, having negative consequences for the whole body system. Circulating lipopolysaccharides (LPSs) can reach the ovarian follicular fluid and can affect the ovaries’ neuroendocrine axis, both of which have negative consequences for the RP of dairy cows [11,165]. Circulatory LPSs are shown to suppress the GnRH and LH activity, and can also reduce the synthesis of PGF2 alpha [166,167,168]. Fluctuations in feeding behavior characterized by low feeding time and high intake per feeding are the contributing factors in the occurrence of ruminal acidosis [169]. Since maintaining proper nutritional support and gut health is very important in the speedy recovery of postpartum reproductive health and ovarian cyclicity, ruminal acidosis should be a greater concern for the postpartum cow. Earlier we discussed that feeding UFAs is helpful for postpartum energy balance and reproduction, and their feeding can have negative impact on rumen microbiota [170]. This negative impact can be minimized in diets with the inclusion of high forage content, which maximizes ruminal biohydrogenation [171]. On the other hand, essential consumption of a concentrate-rich diet acts conversely by decreasing biohydrogenation [171,172]. Therefore, prepartum feeding adjustments to incorporate all the ingredients contained in the postpartum feed is advised to avoid ruminal acidosis and successful energy-rich feed adaptation of postpartum cows [173]. Probiotics of yeast origin (*Saccharomyces cerevisiae* and *Aspergillus orizae*) are shown to modulate rumen function, avert risk of acidosis, and improve fertility in postpartum cows [173,174]. In conclusion, a careful increase in concentrates with due vigilance for the risk of acidosis and feeding dietary bicarbonates seem to be important in overcoming the problem of ruminal acidosis [175,176].

## 7. Effect of High-Protein Diet on Reproductive Performance

Modern dairy cows produce high milk quantities due to continuous genetic improvement. In order to fulfill the high energy and protein demand of lactation, the protein levels in the cow diet are also increasing. A high proportion of dietary protein, though helpful for lactation yield [177,178], is negatively associated with RP [179]. The proteins in the diet are divided into rumen degradable and non-degradable proteins. Higher rumen degradable protein feeding can disturb the nitrogen cycle, leading to high ammonia and subsequent increases in the blood urea content. Indeed, a high level of rumen non-degradable protein cannot be completely digested in the jejunum and thus should be degraded by microbial flora in the large intestine and transformed into ammonia. The phenomenon could be further promoted by a low level of non-structural carbohydrates in the diet [180]. A meta-analysis found 43% lower odds of reproduction success in cows where plasma urea nitrogen was 19.3 mg/dL or where urea was ≥420 mg/L in the milk compared with lower urea values, where high concentrations of urea nitrogen were negatively associated with reproductive capacity and affect the pituitary and ovarian function of cows and uterine physiology [181]. Studies report a negative correlation of milk urea content of postpartum cows with first postpartum service and conception rates. Another study showed that an increase in milk urea content from 12.5 mg/dL to 13.5 mg/dL caused a 5% decrease in the fertility rate of cows [182]. A high-protein diet and its metabolism generate oxidative stress in the body, affecting reproductive performance [183,184]. Oxidative stress, immune response, heat stress, and changes in gluconeogenesis deplete amino acids, decreasing the availability of essential amino acids [185,186,187]. Extensive protein metabolism may possess negative implications for reproductive performance and lead to depletion of amino acids for oxidative purposes but certain amino acids are essential for reproductive health and pregnancy [188,189]. Therefore, a balanced protein diet and supplementation of certain amino acids such as lysine, arginine, phenylalanine, and tyrosine may be useful for enhancing metabolic status and fertility outcomes of postpartum cows [185,190,191]. Hence, in this context, feeding controlled crude protein and supplementing cotton seed in feed can have better impacts on fertility [192]. Controlled rumen degradable and non-degradable proteins help to provide essential amino acid support and prevent their catabolism [193].

**Figure 2:**
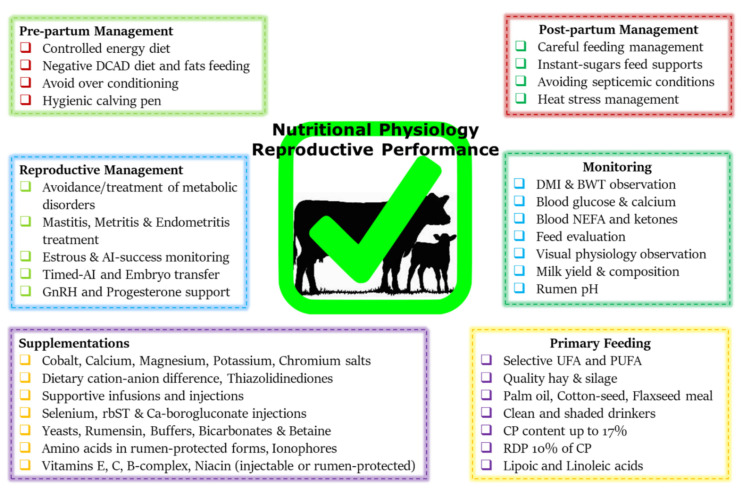
This summary chart encompasses nutritional mitigation and postpartum reproductive management support strategies. Postpartum management should be based upon the advice of dairy extension workers and/or veterinarians, with special care for appropriate feeding practices. (Abbreviations: DCAD, dietary cation–anion difference; DMI, dry matter intake; BWT, body weight; UFA, unsaturated fatty acid; PUFA, poly-unsaturated fatty acid; CP, crude protein; RDP, rumen-degradable protein; inj., injection; rbST, generic somatotropin; AI, artificial insemination; GnRH, gonadotrophin.) This figure is based upon the mitigation charts of our previous studies [9,187] and the recommendations given in this study.

Before concluding this review, a summary chart comprising the nutritional mitigation and postpartum reproductive performance enhancement approaches is presented in Figure 2. This chart is based upon the recommendations discussed in this study and our previous studies [9,187]. Prepartum feeding of a controlled energy diet can help to avoid postpartum NEBAL and improve fertility [68,70], where the inclusion of short-chopped wheat straw or low-quality grass hays can be helpful in this regard [71,194]. The supplementation of the most limited methionine and lysine through rumen-protected means is shown to improve postpartum protein metabolism, improve ovarian follicular biochemical profile [195], conception rate [196,197], and embryonic development [198]. Dietary fat and trace mineral supplementations are also helpful in this regard [199,200]. Monensin supplementation significantly reduces the incidence of subclinical ketosis [201]. Injections of growth hormones such as recombinant bovine somatotropins (rbSTs) are also helpful in improving the metabolic profile and immunity of postpartum dairy cows [202,203]. Besides these nutritional support strategies, the practice of timed AI protocols is demonstrated to be helpful in improving the reproductive outcome of postpartum cows [204,205]. Adoption of synchronized AI protocols effectively makes up for the endocrine alterations and improves follicular ovarian dynamics and thus is effective for breeding success. Employment of embryo transfer technology can effectively bypass the initial reproductive processes and is helpful for enhanced reproductive outcomes [206]. Furthermore, injecting post-AI GnRH and progesterone is also helpful to support the corpus lutea and increase conception rates [207,208].

## 8. Conclusions

Postpartum metabolic disorders debilitate dairy cows and predispose them to a decline in postpartum reproductive efficiency. Perinatal NEBAL appears to be the major culprit behind the occurrence of post-parturient metabolic diseases and related conditions. Prepartum BCS and postpartum lipid mobilization are the two factors closely associated with NEBAL. A controlled energy diet starting from −21 d is helpful in successful perinatal transition of dairy cows. While doing so, attention must be paid to avoid over-conditioning during the prepartum period. There is enough literature about the management of perinatal cows to avoid the occurrence of NEBAL and excessive NEFA mobilization. However, controversy still exists about the supplementations of fats, their type, and quantity fed. Therefore, further research involving complex farm trials about fat supplementation would help move in the right direction. NEFA and ketone interactions with the ovarian–hypothalamus–pituitary axis, oocytes, and developing embryos at the system biology level can bring up exciting knowledge and insights. A score of nutritional mitigation strategies are available and future discoveries will help to maximize the welfare and reproductive efficiency of postpartum dairy cows.
